# Prevalence, Metabolic Profile, and Associated Risk Factors of Non-alcoholic Fatty Liver Disease in an Adult Population of India

**DOI:** 10.7759/cureus.33977

**Published:** 2023-01-19

**Authors:** Abhishek Singhai, Vikas Yadav, Rajnish Joshi, Rajesh Malik, Savitha B T, Sarita Kamle

**Affiliations:** 1 General Medicine, All India Institute of Medical Sciences, Bhopal, Bhopal, IND; 2 Environmental Health and Epidemiology, Indian Council of Medical Research-National Institute for Research in Environmental Health, Bhopal, IND; 3 Internal Medicine, All India Institute of Medical Sciences, Bhopal, Bhopal, IND; 4 Radiodiagnosis, All India Institute of Medical Sciences, Bhopal, IND; 5 General Medicine, All India Institute of Medical Sciences, Bhopal, IND

**Keywords:** liver cirrhosis, type 2 diabets mellitus, community obesity, metabolic diseases, nonalcoholic fatty liver disease (nafld)

## Abstract

Introduction

Non-alcoholic fatty liver disease (NAFLD) is the main cause of chronic liver disease worldwide. NAFLD refers to a group of diseases that includes simple steatosis, nonalcoholic steatohepatitis, cirrhosis, and hepatocellular carcinoma. Unfortunately, there aren't many studies on NAFLD conducted in India. The majority of research involved specific populations, such as diabetics, pregnant women with gestational diabetes, and obese or non-obese people. When the current study was being planned, there were few population-based studies available. In almost all of the research, ultrasound was employed to identify NAFLD, and the whole spectrum of NAFLD was not assessed. The full spectrum of NAFLD in India must have been considered, including all stages of steatosis as well as hepatic damage as shown by high alanine aminotransferase levels and fibrosis. The purpose of this study was to determine the prevalence, spectrum, and metabolic determinants of NAFLD as assessed by FibroScan® (FibroScan® expert 630 machine; Echosens, Paris, France) in adults of Central India.

Methods

This cross-sectional study was conducted among 236 adults aged 18 years and above in three localities of Bhopal, India from March 2022 to October 2022. The study included males and females who provided informed consent and fulfilled inclusion criteria. One research assistant and one staff nurse solicited people to participate in the FibroScan® test during the community screening and shared information about the programme. All participants were subjected to the FibroScan® test.

Results

A total of 322 healthy adults were approached for possible inclusion in the study. Data from 236 subjects were available for analysis after meeting the inclusion and exclusion criteria. According to this study, 43.6% of the study population had NAFLD as detected by FibroScan®. Out of the total, 12.7% of subjects had steatosis grade 1 (S1), 12.3% of subjects had steatosis grade 2 (S2), and 18.6% of subjects had steatosis grade 3 (S3). High body weight, high waist circumference, high waist-to-hip ratio, high fasting sugar, high serum glutamate pyruvate transaminase (SGPT), high triglyceride levels and high very low-density lipoprotein (VLDL) levels were significantly associated with NAFLD.

Conclusion

In conclusion, 43.6% of the adult population of Bhopal, India is suffering from NAFLD. NAFLD is a severe burden in the Indian community despite being historically associated with the western world. Obesity, diabetes and dyslipidemia are significantly associated with NAFLD.

## Introduction

One-fourth of the world's population is affected by non-alcoholic fatty liver disease (NAFLD) [1]. Simple steatosis, nonalcoholic steatohepatitis (NASH), cirrhosis, and hepatocellular carcinoma (HCC) are all included in the NAFLD group of disorders [2,3]. In the entire world, it is the main cause of chronic liver disease. It is the fastest-growing indication for liver transplantation in the United States, the United Kingdom, and several low-and middle-income countries [4,5]. The most prevalent chronic liver disease worldwide is NAFLD [6]. NAFLD is now considered a hepatic element of metabolic syndrome (MS) and has been associated with obesity, insulin resistance, type 2 diabetes, dyslipidemia, hypertension, and cardiovascular disease [7].

The Middle East and South America have the greatest rates of NAFLD in adults over the age of 18, followed by Asia, North America, Europe, and Africa [8]. The estimated global prevalence of NAFLD in individuals over the age of 18 is 25.2%. According to a recent meta-analysis, Asia has shown a sharp increase in the prevalence of NAFLD. It revealed a pooled frequency of 29.6% in Asia, with significant regional heterogeneity [9]. The significance of NAFLD for public health derives from its global effects on morbidity, death, and the use of medical services.

NASH and hepatic fibrosis are linked to higher rates of death from all causes, cardiovascular disease, and liver disease in the general population. Even in populations with low body mass index (BMI), like India, the excessive cardiovascular risk associated with non-alcoholic fatty liver has been documented [10].

In a newly released systematic review and meta-analysis of 111 papers, the prevalence of MS in the adult population of India was estimated to be 30% (95% CI: 28%-33%). The frequency was significantly higher with age, in institution studies, in cities, and in women, but there were no discernible regional or temporal changes between 2003 and 2019 according to the authors [10].

Unfortunately, there aren't many studies on NAFLD in the Indian population. The majority of research involved specific populations, such as diabetics, pregnant women with gestational diabetes, obese or non-obese people, and so forth [11,12]. When the current study was being planned, there were few population-based studies available, and they indicated a prevalence of NAFLD ranging from 19% to 32.0% [13,14]. In almost all of the research, ultrasound was employed to identify NAFLD, and the whole spectrum of NAFLD was not assessed [15]. The full spectrum of NAFLD in India must have been considered, including all stages of steatosis as well as hepatic damage as shown by high AST/ALT levels and fibrosis. The aim of this study was to identify the prevalence, spectrum, and metabolic determinants of NAFLD in Indian adults.

## Materials and methods

Study design

From March 2022 to October 2022, this cross-sectional survey was carried out among persons in three Bhopal localities who were 18 years of age and older. The study included males and females who provided informed consent and fulfilled the inclusion criteria. The study excluded people who were consuming a significant amount of alcohol or who had overt cirrhosis, previously diagnosed Hepatitis B virus or Hepatitis C virus infection, biliary diseases, and pregnant females.

**Figure 1:**
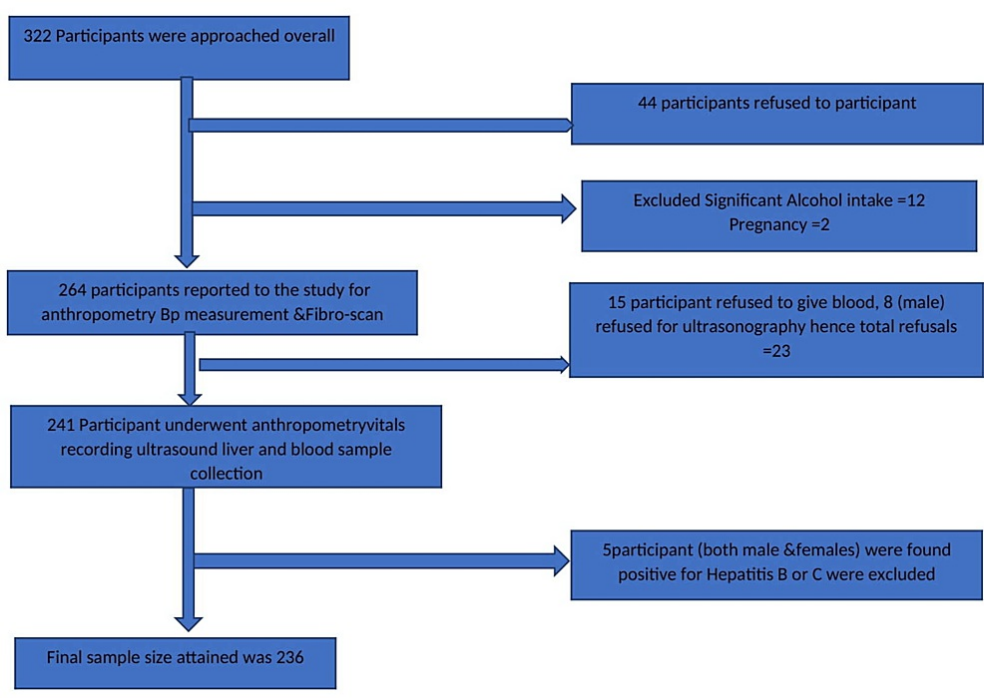
Flow chart showing the process of subjects recruitment

During the community screening, one research assistant and one staff nurse raised awareness about the programme and invited people for FibroScan® testing. FibroScan® is a non-invasive device that uses transient elastography to measure the liver's "hardness" (or stiffness). Figure 1 displays the study protocol. The screening was done by recording medical history to include the desired study population. Anthropometric measurements were taken by trained research assistants, which included height (cm), weight (kg), waist circumference (cm), hip circumference (cm), waist-to-hip ratio, and BMI. The staff nurse measured blood pressure (BP) in a sitting position. After an overnight fast, an ultrasound of the liver and biochemical tests (fasting blood sugar, lipid profile, serum glutamate pyruvate transaminase (SGPT), Hepatitis B surface antigen (HBsAg), anti-Hepatitis C virus antibodies (HCV) were performed. Before the interview and all other examinations, study participants provided written informed consent.

Study procedure

Every eligible participant underwent a FibroScan® test. The FibroScan® is a non-invasive device that uses transient elastography to measure the liver's "hardness" (or stiffness). By monitoring the speed of a vibration wave (also known as a "shear wave") produced on the skin, liver hardness can be assessed. The amount of time it takes a vibration wave to travel to a specific depth within the liver is used to calculate the shear wave velocity. The degree of hepatic fibrosis can be determined by the liver's hardness since fibrous tissue is harder than normal liver. A minimum of 10 valid readings with a success rate of at least 60% and an interquartile range of 30% of the median value were taken in order to increase test reliability. The results were expressed in kilopascals (kPa). The FibroScan® test is completely non-invasive, easy to perform, and painless. It takes about 10 minutes. On an exam table, patients were instructed to lie completely flat. On the right side of the lower chest wall (6th or 7th intercostal space), a technician positioned the FibroScan® (FibroScan® expert 630 machine; Echosens, Paris, France) probe between the ribs. The liver was next given a series of painless pulses.

A total score for the liver stiffness measurement (LSM) was produced after the data were recorded on the apparatus. A skilled physician then used this number to forecast the possibility of fibrosis or fatty liver. FibroScan® measures LSM score in kilopascals and controlled attenuation parameter (CAP) score in decibels per metre (dB/m). A CAP score can be between 100 and 400 dB/m. Table 1 displays the CAP score ranges together with the corresponding steatosis grade and degree of fatty alteration.

**Table 1:** Table showing CAP score ranges together with the corresponding steatosis grade and degree of fatty alteration. CAP: Controlled attenuation parameter, dB/m: decibels per meter

CAP Score	Steatosis Grade	Amount of Liver with Fatty Change
238 to 260 dB/m	S1	11% to 33%
260 to 290 dB/m	S2	34% to 66%
Higher than 290 dB/m	S3	67% or more

The liver's degree of scarring can be determined by looking at fibrosis results. In healthy people, the LSM score falls between 2 and 6 kPa, 75 kPa is the greatest outcome that is feasible. The scarring has four stages: F0 to F1 fibrosis score (No or little liver damage), F2 fibrosis (Moderate liver scarring), F3 fibrosis (severe liver scarring), and F4 fibrosis (cirrhosis). Table 2 lists several liver conditions, fibrosis outcome ranges, and the corresponding LSM score.

**Table 2:** Table showing liver diseases, ranges of fibrosis results, and the matching LSM score LSM: Liver stiffness measurement, kPa: kilopascals

	F0 to F1	F2	F3	F4
Hepatitis B	2 to 7 kPa	8 to 9 kPa	8 to 11 kPa	18 kPa or higher
Hepatitis C	2 to 7 kPa	8 to 9 kPa	9 to 14 kPa	14 kPa or higher
HIV/HCV Coinfection	2 to 7 kPa	7 to 11 kPa	11 to 14 kPa	14 kPa or higher
Cholestatic Disease	2 to 7 kPa	7 to 9 kPa	9 to 17 kPa	17 kPa or higher
Non-Alcoholic Fatty Liver Disease (NAFLD)	2 to 7 kPa	7.5 to 10 kPa	10 to 14 kPa	14 kPa or higher
Alcohol-Related Disease	2 to 7 kPa	7 to 11 kPa	11 to 19 kPa	19 kPa or higher

NAFLD subjects were further subdivided into the following subgroups based on the FibroScan® parameters like CAP and LSM score (Table 3).

**Table 3:** Table showing a diagnosis of stages of NAFLD based on CAP score and LSM score NAFLD: Non-alcoholic fatty liver disease, CAP: Controlled attenuation parameter, LSM: Liver stiffness measurement

Disease condition	CAP score (dB/m)	LSM score (kPa)
Nonalcoholic Fatty liver	>238	<7
Nonalcoholic steatohepatitis	>238	7.1-14
Nonalcoholic cirrhosis	>238	>14

Ethical committee review

All procedures were followed according to the guidelines of the Indian Council of Medical Research (ICMR) and All India Institute of Medical Sciences (AIIMS), Bhopal, to protect the rights of the patients. Approval for the study has been obtained from the Institutional Ethics Committee of AIIMS, Bhopal, before the start of the study (approval number: IHEC-LOP/2020/ EF0193).

Statistical analysis

Microsoft Excel (Microsoft Corp., Redmond, WA) was used for data entry, and SPSS software, version 25.0 (IBM Corp., Armonk, NY) was used for data processing. If a numerical variable has a regularly distributed distribution, it is expressed as the mean +/- SD; otherwise, it is written as the median and interquartile range. Number percentages were used to summarise categorical variables. Using the Chi-square test, associations between categorical variables were examined.

## Results

Figure 1 shows a flow chart of the participant selection process. A total of 322 healthy adults were approached for possible inclusion in the study; 44 adults refused to participate, 12 adults were excluded due to significant alcohol intake, and two pregnant females were also excluded from the study. Therefore, 264 adults were screened for fatty liver by FibroScan®. Further 15 adults refused blood tests, eight refused ultrasonography, and five were excluded due to HbsAg or anti-HCV positive status. So finally data from 236 subjects were available for analysis.

Table 4 shows the sociodemographic characteristics of the study participants. The mean (+SD) age of the subjects was 33.5 (+9) years. The majority of our subjects were young, 81.4% of participants were less than 50 years old. Out of the total subjects, 72 % were male. The mean BMI of subjects was 24.62 (+4.41). The mean (+SD) waist circumference of subjects was 91.15 (+11.20) cm. The mean (+SD) hip circumference of subjects was 99.11 (+11.07) cm. The mean (+SD) waist-to-hip ratio was 0.91 (+0.07). The mean (+SD) CAP score of subjects was 240.30 (+48.13) dB/m. The mean (+SD) fasting blood sugar (FBS) of subjects was 94.60 (+25.84) mg/dl. The mean (+SD) SGPT of subjects was 32.78 (+35.35) U/L. The mean (+SD) serum cholesterol of subjects was 152.29 (+34.46) mg/dl. The mean (+SD) serum triglyceride of subjects was 124.97 (+78.08) mg/dl. The mean (+SD) high-density lipoprotein (HDL), low-density lipoprotein (LDL), and very low-density lipoprotein (VLDL) of subjects were 39.84 (+15.40), 88.30 (+28.14), and 25.72 (+17.81) mg/dl respectively. The study participant’s personal history revealed that six subjects had hypertension, four subjects had diabetes and one subject had hypothyroidism.

**Table 4:** Table showing sociodemographic characteristics of the study participants CAP: Controlled attenuation parameter, FBS: Fasting blood sugar, SGPT: Serum Glutamic Pyruvic Transaminase, HDL: High-density lipoprotein, LDL: Low-density lipoprotein, VLDL: Very low-density lipoprotein, Cm: Centimeter, Kg: Kilogram, Kg/M^2^: Kilogram/meter^2^, dB/m: Decibel per meter, U/L: Unit per litre, mg/dl: Milligrams per decilitre

Descriptive Statistics					
	N	Minimum	Maximum	Mean	Std. Deviation
Age (Years)	236	18.00	62.00	33.5254	9.03017
Height (Cm)	236	145.00	191.00	165.3305	8.96523
Weight (Kg)	236	35.00	142.00	68.6737	14.03247
BMI (Kg/M^2^)	236	15.00	49.00	24.6229	4.40958
Waist circumference (Cm)	236	52.00	140.00	91.1568	11.20965
Hip circumference (Cm)	236	54.00	187.00	99.1144	11.07151
Waist-to-hip ratio	236	0.48	1.27	0.9154	0.07653
CAP score (dB/M)	236	131.00	382.00	240.3093	48.13670
FBS (mg/dl)	236	50.00	282.00	94.6017	25.84195
SGPT (U/L)	236	6.00	455.00	32.7839	35.35726
Serum cholesterol (mg/dl)	236	33.00	245.00	152.2924	34.46138
Serum triglycerides (mg/dl)	236	34.00	794.00	124.9788	78.08018
HDL (mg/dl)	236	20.00	196.00	39.8475	15.40634
LDL (mg/dl)	236	9.00	179.00	88.3051	28.14340
VLDL (mg/dl)	236	7.00	158.00	25.7288	17.81855
Valid Numbers	236				

According to this study, 43.6% of the adult population had NAFLD as detected by FibroScan®, 12.7% subjects had steatosis grade 1 (S1), 12.3 % subjects had steatosis grade 2 (S2), and 18.6% subjects had steatosis grade 3 (S3).

Table 5 compares subjects with NAFLD (high CAP score) and without NAFLD (low CAP score). The mean age of NAFLD cases was 34.91 years and the mean age of controls was 32.45 years. The mean BMI of NAFLD cases was 26.45 while that of controls was 23.20. The mean waist-to-hip ratio of NAFLD cases was 0.93 while that of controls was 0.89. The mean FBS of NAFLD cases was 100.18 mg/dl while that of controls was 90.27 mg/dl. The mean SGPT of NAFLD cases was 38.03 U/L while that of controls was 28.71 U/L. Mean serum cholesterol, triglycerides, HDL, LAD, and VLDL of NAFLD cases were 152.92, 139.14, 39.26, 90.23, and 28.41 mg/dl respectively while these parameters of controls were 151.80, 114, 40.30, 86.81 and 23.64 mg/dl respectively. High body weight, high waist circumference, high waist-to-hip ratio, high fasting sugar, high SGPT, high triglyceride levels, and high VLDL levels were significantly associated with NAFLD.

**Table 5:** Table comparing features of subjects with NAFLD (high CAP score) and without NAFLD (low CAP score) CAP: Controlled attenuation parameter, FBS: Fasting blood sugar, SGPT: Serum Glutamic Pyruvic Transaminase, HDL: High-density lipoprotein, LDL: Low-density lipoprotein, VLDL: Very low-density lipoprotein, Cm: Centimeter, Kg: Kilogram, Kg/M^2^: Kilogram/meter^2^, dB/m: Decibel per meter, U/L: Unit per litre, mg/dl: Milligrams per decilitre

Group Statistics									
		N	Mean	Mean Difference	Std. Deviation	Std. Error Mean	t test value	df	p value
Age (Years)	Low CAP	133	32.4511	-2.46149	8.67727	0.75241	-2.092	234	0.038
	High CAP	103	34.9126		9.32644	0.91896			
Height (Cm)	Low CAP	133	164.6541	-1.54975	8.83266	0.76589	-1.319	234	0.188
	High CAP	103	166.2039		9.10221	0.89687			
Weight (Kg)	Low CAP	133	64.1805	-10.29528	11.52062	0.99896	-5.990	234	0.000
	High CAP	103	74.4757		14.88574	1.46674			
BMI (Kg/M^2^)	Low CAP	133	23.2030	-3.25330	3.60505	0.31260	-6.029	234	0.000
	High CAP	103	26.4563		4.68577	0.46170			
Waist circumference (Cm)	Low CAP	133	87.0000	-9.52427	9.58455	0.83109	-7.126	234	0.000
	High CAP	103	96.5243		10.90857	1.07485			
Hip circumference (Cm)	Low CAP	133	96.5338	-5.91277	11.36818	0.98575	-4.211	234	0.000
	High CAP	103	102.4466		9.76059	0.96174			
Waist to hip ratio	Low CAP	133	0.8977	-0.04051	0.07932	0.00688	-4.171	234	0.000
	High CAP	103	0.9383		0.06646	0.00655			
FBS (mg/dl)	Low CAP	133	90.2782	-9.90627	19.09163	1.65545	-2.969	234	0.003
	High CAP	103	100.1845		31.79677	3.13303			
SGPT (U/L)	Low CAP	133	28.7143	-9.32455	40.44554	3.50707	-2.022	234	0.044
	High CAP	103	38.0388		26.71635	2.63244			
Serum cholesterol (mg/dl)	Low CAP	133	151.8045	-1.11782	33.59551	2.91310	-0.247	234	0.805
	High CAP	103	152.9223		35.70404	3.51802			
Serum triglycerides (mg/dl)	Low CAP	133	114.0075	-25.13811	60.10086	5.21141	-2.480	234	0.014
	High CAP	103	139.1456		94.93036	9.35377			
HDL (mg/dl)	Low CAP	133	40.3008	1.03862	13.48680	1.16945	0.513	234	0.609
	High CAP	103	39.2621		17.63072	1.73721			
LDL (mg/dl)	Low CAP	133	86.8120	-3.42098	28.83263	2.50011	-0.926	234	0.355
	High CAP	103	90.2330		27.24587	2.68462			
VLDL (mg/dl)	Low CAP	133	23.6466	-4.77086	14.35597	1.24482	-2.054	234	0.041
	High CAP	103	28.4175		21.25638	2.09445			

## Discussion

This cross-sectional study was carried out in three localities of Bhopal, India. In our study prevalence of NAFLD was 43.6%. NAFLD risk factors included obesity, increased blood fasting sugar, and dyslipidemia. From 8.7% to 57.37% of people in Asia have NAFLD [16,17]. Between 16.6% and 32% of the urban population in India suffers from NAFLD [18].

Larger waist circumference subjects were considerably more likely to develop NAFLD. The results were consistent with past research that showed strong associations between NAFLD and this parameter [19]. In this study, it was found that abdominal obesity could predict NAFLD on its own. Even if their BMI is the same as that of the Western population, Asians are more likely to have abdominal fat deposition and are more likely to develop obesity-related diseases due to excess visceral fat [20]. Despite having lower BMIs, Asians are more vulnerable to DM and cardiovascular risk factors because of their central obesity. Despite having lower BMIs, Asians have a higher prevalence of NAFLD, which may be caused by an abnormal deposition of visceral fat.

NAFLD and high fasting sugar were significantly correlated. Other researchers also suggested that NAFLD and high fasting sugar are related [4]. Despite being a known risk factor in other research, high serum cholesterol levels were not an independent risk factor in the current investigation. Participants with high triglycerides and high VLDL had a significantly greater risk of developing NAFLD. This was comparable to earlier work that demonstrated significant relationships between NAFLD and this parameter [8].

Studies have indicated that people with NAFLD, both with and without diabetes, have a higher frequency of cardiovascular disease (CVD) [21,22]. NAFLD is therefore typically linked to an unhealthy lifestyle, and there is evidence to support the idea that making changes to an unhealthy lifestyle can lower transaminase levels and ameliorate NAFLD [23]. Beyond the usual CVD risk factors, medication use, and diabetes-related variables, a study of patients with T2DM found that the prevalence of peripheral vascular, coronary, and cerebrovascular diseases was higher in subjects with NAFLD than in subjects without this disease. This was true for both coronary, cerebrovascular, and peripheral vascular diseases. According to Byrne et al., there have been over 20 studies that have been published, both prospective and retrospective, examining the connection between NAFLD and cardiovascular disease, and they have come to the conclusion that NAFLD is a definite risk factor for CVD [24], which is further supported by ongoing studies [25].

There are a few limitations of our study also. This study was planned in one city only so generalizability is questionable. Participation was voluntary, so some cases may have been missed. As this was a community-based screening study, extensive investigations to exclude other rarer causes of liver disease could not be performed.

## Conclusions

In conclusion, 43.6% of the study population of Bhopal is suffering from NAFLD. A large number of subjects (18.6%) had severe degrees of fatty changes (S3). NAFLD is a severe burden in the Indian community, despite it being historically associated with the western world. Obesity, diabetes, and dyslipidemia are just a few of the MS's accompanying conditions that constitute a significant burden. To determine the prevalence of fibrosis and cirrhosis in the population, more research is required. Additionally, public health interventions are required to regulate, prevent, and treat NAFLD, MS, and their negative health effects.
